# The Dynamics of Circulating Immune Complexes in Horses with Severe Equine Asthma

**DOI:** 10.3390/ani11041001

**Published:** 2021-04-02

**Authors:** Malwina Slowikowska, Joanna Bajzert, Julia Miller, Tadeusz Stefaniak, Artur Niedzwiedz

**Affiliations:** 1 Department of Internal Medicine and Clinic of Diseases of Horses, Dogs and Cats, Faculty of Veterinary Medicine, Wroclaw University of Environmental and Life Sciences, 50-366 Wroclaw, Poland; artur.niedzwiedz@upwr.edu.pl; 2 Department of Immunology, Pathophysiology and Veterinary Preventive Medicine, Faculty of Veterinary Medicine, Wroclaw University of Environmental and Life Sciences, 50-375 Wroclaw, Poland; joanna.bajzert@upwr.edu.pl (J.B.); julia.miller@upwr.edu.pl (J.M.); tadeusz.stefaniak@upwr.edu.pl (T.S.)

**Keywords:** circulating immune complexes, equine asthma syndrome, heaves, recurrent airway obstruction, asthma environmental challenge

## Abstract

**Simple Summary:**

Equine asthma syndrome is a cost-consuming equine respiratory disease of the lower airways in horses. Non-invasive biomarkers from blood or urine are sought. The aim of this study was to assess the circulating immune complexes (CICs) during the exacerbation and remission of an asthma episode—with and without additional treatment and the potential usefulness of CIC levels in the diagnosis, monitoring, and treatment progression. The control group, asthma group, and treated asthma group each contained six horses. The horses were kept in a dusty environment for seven days and then moved to an asthma-friendly environment for over three weeks (the treated group received injections of glucocorticoids). Blood was collected at baseline and on the 1st, 2nd, 3rd, 7th, 14th and 30th days. CICs measured in the time points did not show statistical differences. When CICs were analysed within the groups, there was a significant decrease in CIC in the treated group and a significant increase in CIC in the non-treated group on day 30. CIC did not support the diagnosis procedure of equine asthma syndrome, although it may help in monitoring patients with and without treatment. To the best of the authors’ knowledge, this is the first study to analyse the dynamics of CIC in equine asthma patients during an environmental challenge, remission, and treatment.

**Abstract:**

Non-invasive diagnostic biomarkers of equine asthma syndrome (EAS) from blood or urine are sought. The aim of this study was to assess the absorbance of circulating immune complexes (CICs) during the exacerbation, remission, and treatment of an asthma episode and assess the potential usefulness of CIC levels in the diagnosis and monitoring of the disease. The control group, asthma group, and treated asthma group each contained six horses. Following an initial examination and group classification, the horses were kept in a dusty environment for seven days and then moved to an asthma-friendly environment for three weeks (the treated group received injections of glucocorticoids). Blood was collected at baseline and on the 1st, 2nd, 3rd, 7th, 14th and 30th days. CIC was measured using the modified Haskova method. The time points did not show significant statistical differences. There was a significant decrease in CIC in the treated group, and a significant increase in CIC in the non-treated group on day 30. CIC did not support the EAS diagnosis, although it may help in monitoring patients. To the best of the authors’ knowledge, this is the first study to analyse the dynamics of CIC during environmental challenge, remission, and treatment.

## 1. Introduction

Equine asthma syndrome, which appears in light/moderate and severe forms, is most commonly diagnosed in a temperate climate [1,2]. Severe equine asthma usually affects adult horses and is associated with frequent coughing, exercise intolerance, and increased respiratory effort at rest [2]. Estimated disease prevalence in the northern hemisphere is between 10% and 20% in the adult horse population [3].

Unlike human asthma, a type 1 hypersensitivity response is not observed after an environmental challenge in asthmatic horses. Higher histamine activity observed in the lungs of horses approximately five hours after exposure to an allergen suggests a type 3 hypersensitivity response [1,3,4]. Researchers have tried to characterise the cytokine profile during an asthma exacerbation. However, the published results are still inconsistent, especially those regarding the expression of INF-γ, IL-4, IL-5, and IL-13 [5,6,7,8]. Although the immunological pathway of this disease has not been fully determined, the significant contribution of the T cells in the pathogenesis of the disease has been confirmed. Results regarding the predominant participation of either Th1 or Th2 remain inconsistent. It is assumed that this may be due to a mixed Th1/Th2 response or a Th1/Th2-imbalance [1,8].

Nevertheless, a major consequence of T cell activation is neutrophil recruitment into the airways [1]. The subsequent immunological and inflammatory processes lead to structural changes in the bronchi and, consequently, to bronchial narrowing. This, in turn, is caused by mucus accumulation, bronchial constriction, and bronchial wall thickening, instigated by smooth muscle hyperplasia and mucosal oedema from inflammatory cell infiltration [1]. These changes also affect the alveolar lumen, leading to impaired gas exchange [9].

Equine asthma syndrome requires a comprehensive clinical approach based on a detailed history, a thorough clinical examination, and some ancillary tests. Due to quite invasive disease diagnostics, especially in severely asthmatic patients, there is a great need for biomarkers to facilitate a diagnosis based on blood analysis. Lately, researchers have also focused on exhaled breath gas and exhaled breath condensate (EBC) and its chemical and metabolomic analysis [10,11,12].

Researchers are focusing more on research based on blood analysis, because it is material that is quite easily obtained in field practice. The formation of circulating immune complexes (CICs) is caused by a basic defence response against endo- and exogenous antigens. The CIC aims to eliminate antigens and present them to immunologically competent cells. CIC detection allows the assessment of the immunological status of patients [13,14]. In human medicine, the CIC relationship with many diseases has been demonstrated, e.g., in allergies, autoimmune diseases, cancers, cardiological, dermatological, endocrinological, nephrological, neurological, ophthalmological, pulmonological, reproductive, haematological disorders, infectious and parasitic diseases [13]. Researchers have again come back to CIC analysis in patients with respiratory diseases. It has been published that there is a significant increase in the concentration of CIC in the blood of asthmatic patients (with atopic bronchial asthma) compared with controls [15], and also in COPD patients [16].

In the available literature, there are few studies on circulating immune complexes in horses. In recent years, research has been published on the concentration of CICs and antigen masses on a group of 42 healthy horses [17]. Some studies concerned horse orthopaedics and foal diseases [18,19]. A preliminary study on the concentration of these complexes in horses with asthma was also published, and showed statistically higher values of CIC concentrations in sick horses compared to healthy ones [20]. These preliminary studies were carried out on a very homogeneous and normogenic group of horses, with uniform environmental conditions and in the permanent habitat of these animals. The results were so interesting that the authors decided to perform the research on a more diverse group of animals from different environments.

The aim of this study was to assess the serum absorption of circulating immune complexes during the exacerbation and remission of an asthma episode in asthmatic patients with and without additional treatment. In addition, the usefulness of CIC levels in monitoring the disease progression and treatment was assessed.

## 2. Materials and Methods

A consent of the Wrocław II Local Ethic Committee was obtained for this study (resolution no. 45/2014 from 2 April 2014, and consent for experiment changes with resolution no. 47/2015 from 17 June 2015). The owners were informed about the study design and the risks, the owners’ consent was obtained.

### 2.1. Materials

Eighteen horses were included in the study. The horses were divided into three groups: the control group, untreated asthma patients, and treated asthma patients. The control group (group 1) contained six healthy adult horses: four mares and two geldings, from 4 to 9 years of age. Groups 2 and 3 contained animals diagnosed with severe equine asthma. Group 2 included six adult horses that did not undergo treatment during the study period. There were three mares and three geldings, from 11 to 18 years of age in the group. One of the mares was euthanised due to severe colic with no consent for surgical approach from the owner. Group 3 contained six adult horses, from 4 to 8 years of age, four mares and two geldings. Those animals were treated pharmacologically during the experiment.

Only animals with no known comorbidities or pathological symptoms other than asthma (for the asthma groups) were qualified in the study.

### 2.2. Methods

#### 2.2.1. Clinical Experiment Part

##### Preliminary Procedure

Each animal underwent a full preliminary procedure, to determine whether the horse was clinically healthy or had severe equine asthma. The animals were firstly qualified for preliminary procedures by the patient history and clinical symptoms. After the full preliminary procedures, only animals that clearly qualified as healthy or with severe equine asthma were included in the study. This procedure consisted of a detailed history, a clinical examination, basic blood tests, arterial blood gasometry, respiratory endoscopy and bronchoalveolar lavage. Samples of 25 mL of venous blood were obtained from the jugular vein using a 1.2 mm needle (18 G) and a 25 mL syringe. The arterial blood samples were obtained from the transverse facial artery with a 0.8 mm (21 G) butterfly needle and a heparinized 2 mL syringe. The arterial blood gas analysis (saturation, partial pressure of oxygen and carbon dioxide) was performed immediately after blood collection using the Osmotech OPTI Blood Gas Analyser (Roswell, NM, USA).

##### Respiratory Endoscopy and Bronchoalveolar Lavage

The horses were sedated with 0.6–1 mg/kg bw xylazine (Nerfasin vet. 100 mg/mL, LIVISTO Sp. z o.o., Oudewater, Holand) and 25–50 μg/kg bw butorphanol (Morphasol 10 mg/mL, LIVISTO Sp. z o.o., Senden-Bösensell, Germany). The endoscopy was performed using a 180 cm-long video endoscope with an outside diameter of 9 mm (Karl Storz, Tuttlingen, Germany) and a Karl Storz GarstoPack portable light source (Tuttlingen, Germany). During the endoscopy, the appearance of the tracheal mucous membrane, as well as the mucus coloration and amount, were rated. The bronchoalveolar lavage was performed with a special catheter—a bronchoalveolar lavage catheter (Equivet B.A.L., Kruuse, Langeskov, Denmark). A total amount of 300 mL 0.9% sodium chlorate (Baxter, Warsaw, Poland) was passed into the lungs and withdrawn into the syringe. The obtained material (over 50% of the fluid was retrieved from each horse) underwent cytological analysis on the same day, following transportation on ice. The samples were centrifuged for 5 min at 2000× *g*. A smear from the sediment was stained according to the May–Grunwald–Giemsa method.

The cytological assessment was based on a 400-cell leukocyte differential percentage count according to the standard equine asthma diagnostic procedure [21].

The clinical and endoscopic score was analysed using the modified Tilley score [22] (Appendix A, Table A1). Only animals with a final score of 0 were included in the control group according to cough score, nostril flare, abdominal lift, and no or few small blobs of colourless runny mucus. The asthma groups had a score from 2 to 3, with common abnormalities of cough, nostril flare and abdominal lift with marked endoscopic changes such as a large amount of thick white (or yellow) mucus threading in the trachea.

##### Experiment Design

The study lasted for over four weeks. During the first seven days, asthma symptoms were exacerbated in the sick animals by an environmental challenge. The control group was kept under the same conditions as the sick animals. During the environmental challenge, the horses were kept in stables for the majority of the day (20–22 h). Straw was used as bedding, the horses were fed poor quality, dusty hay, and dry concentrated feed. Following the period of symptom exacerbation, the horses were kept in asthma-friendly conditions. Asthma-friendly environmental changes were implemented starting from day 8, and the animals were pastured for the majority of the day (8–12 h). Non-dusty bedding was used, and they were fed good quality hay that had been soaked for 20–30 min and wet concentrate feed. The patients were kept in the asthma-friendly environment for over three weeks. In the third group, the horses also received an intravenous treatment, starting from day 8, with steroid drugs containing dexamethasone sodium phosphate, which was initially administered at 0.096 mg/kg (day 8), followed by a second dose at 0.062 mg/kg (day 9) and a third dose at 0.031 mg/kg (day 10) (Rapidexon 2 mg/mL, DECHRA, Bladcl, The Netherlands).

**Figure 1:**
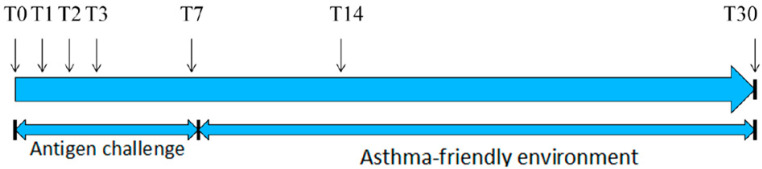
Experiment timeline.

Blood samples were obtained from the external jugular vein at the beginning of the experiment (T0), 24 h after beginning the environmental challenge (T1), and 48 h (T2), 72 h (T3), 7 days (T7), 14 days (T14) and 30 days after commencing the experiment (T30) (Figure 1). Tubes with EDTA, sodium citrate, and serum separation granules were used for sample collection. A basic clinical examination was performed prior to blood collection.

The collected blood was assessed on the day of collection. The remaining blood (both plasma and serum) was centrifuged and divided into 0.6–1.7 mL portions and frozen at −80 °C until further analysis.

#### 2.2.2. Laboratory Tests

##### Haematology and Biochemistry Blood Tests

The venous blood haematology analysis was carried out using the ABC Vet equipment (Northampton, UK). The biochemistry blood test at the baseline was performed using the Konlab PRIME 30i (Thermo Fisher Scientific, Vantaa, Finland). The total bilirubin (BILI-T), urea, creatinine, albumin, total protein, glucose, total calcium, iron, sodium, potassium, chloride, magnesium, bile acids, aspirate aminotransferase (ASPAT), alanine aminotransferase (ALAT), gamma-glutamyl transpeptidase (GGTP), and creatinine kinase (CK) parameters were assessed.

##### Quantification of Circulating Immune Complexes

The detection and quantification of circulating immune complexes was performed using a modified method with polyethylene glycol (PEG) according to Hašková et al. [23]. In order to determine the concentration of immune complexes present in the horse serum, 750 μL of a 3.3% sterile polyethylene glycol (PEG) solution were added to a 250 μL sample. The obtained solution was then incubated at 4 °C for 18 h and centrifuged for 15 min at 24,000× *g* at 4 °C. The obtained sediment was washed with 2.5% PEG and centrifuged for 15 min at 24,000× *g* at 4 °C. The sediment was dissolved in 2 mL of a 0.1% sodium hydroxide solution (NaOH). The optical density was measured at a wavelength of 280 nm using the option of a pathlength correction with a µQuantum ELISA-Microplate reader (BioTek Instruments, Winooski, VT, USA) and the Greiner UV-Star microplate.

##### The Detection of Equine IgG in Circulating Immune Complexes (CICs) Precipitated Using PEG6000

The CICs were precipitated using PEG6000 as described before, in the serum of 6 horses containing different concentrations of CIC—three with elevated (3,4,6) and three with normal levels of CIC (1,2,5). Concentration of CIC in examined samples: No: 1, 0.0192 mg/mL; 2, 0.0378 mg/mL; 3, 0.166 mg/mL; 4, 0.206 mg/mL; 5, 0.016 mg/mL; 6, 0.149 mg/mL.

The precipitated CICs were washed twice with 2 mL of 2% PEG6000 solution and centrifuged again. The precipitates were dissolved with NaOH solution or dissociation buffer. The presence of equine IgG in CIC was confirmed using double immunodiffusion, counter-current immunoelectrophoresis, SDS-PAGE gel electrophoresis, and immunoblotting.

##### Double Immunodiffusion (ID)

CICs precipitated from serum samples with PEG were washed twice and solubilized in NaOH. CICs were added to respective wells cut in 1.2% Agar Oxoid gel diluted in 0.15 M veronale buffer, pH 8.2. The central well was filled with goat anti-equine IgG antiserum (Pro Animali, Ltd.) The plate was incubated in a wet chamber for 24 h at room temperature and for 24 h at 4 °C. Subsequently, the plates were washed in PBS, dried, and stained with amido-black.

##### Counter-Current Immunoelectrophoresis (IEF)

IEF was performed in 1.2% Agar Oxoid gel diluted in 0.15 mol veronale buffer, pH 8.2, at stable voltage (100 V) for 75 min. Then, 20 µL of solubilized CIC was mixed with 10 µL of 0.1% sodium dodecyl sulphate solution and incubated for 10 min and then added to the wells at (−). The goat anti-equine IgG antiserum was added to the wells located at a distance of 11 mm to (+). The plate was than incubated in a wet chamber for 24 h at room temperature, washed with PBS for 24 h at 4 °C, dried, and stained with amido-black.

##### SDS-PAGE Gel Electrophoresis

After centrifugation (15 min; 24,000× *g*; 4 °C), the precipitates (CIC) were dissolved/resuspended in 200 μL of dissociation buffer (1× Laemmli Sample Buffer, BioRad with 5% 2-mercaptoethanol or without 2-mercaptoethanol). Then, the samples were heated at 100 °C for 5 min and centrifuged for 10 min at 10,000× *g*. The CIC samples were separated by SDS-PAGE gel electrophoresis in 7% polyacrylamide gels (~50 μg/lane). At the end of the electrophoretic run, separated proteins were stained with Coomassie Brilliant Blue R 250. Gel documentation was made using ChemiDoc XRS+ Instruments (BioRad). The molecular weights of proteins were estimated using Image LabTM software (BioRad) by comparison with standard protein markers (Spectra Multicolor Broad Range Protein Ladder BR or HR).

##### Immunoblotting Analysis

CIC samples (1, 3, 4, 2) resuspended in 200 μL of dissociation buffer (Laemmli Sample Buffer, BioRad with 5% 2-mercaptoethanol) were separated by SDS-PAGE gel electrophoresis in 7% polyacrylamide gels and transferred onto nitrocellulose membranes (Sigma Aldrich) (using a Trans Blot Turbo, BioRad semi-dry chamber; the transfer conditions: 25 V const., 1.5 mA, 30 min). Membrane was blocked using Western blot blocking buffer (Invitrogen; blocking conditions: 180 min at RT). The membrane was incubated with the goat anti-equine IgGb (Serotec no AAI36P; diluted 1:10,000 in TBST buffer) for 90 min at RT. The reaction was developed using ECL substrate (BioRad). The membrane visualisation was performed using Gel Doc XR Instruments (BioRad). The molecular weights of proteins were estimated using Image LabTM software (version 5.2.1; BioRad) by comparison with standard protein marker (SpectraTM Multicolor Broad Range Protein Ladder, Fermentas).

### 2.3. Statistical Analysis

All statistical analyses were performed using the R statistical package for Windows (version 3.4.3., R Foundation for Statistical Computing, Vienna, Austria) using non-parametric tests [24].

The analysis of qualifying tests and the analysis of the differences in the absorbance of CIC between the groups were carried out using the Kruskal–Wallis test, with a post hoc analysis using the Conover method.

An analysis of the changes over time was conducted using linear mixed models. In addition, time models for the entire population were created.

## 3. Results

### 3.1. Analysis of the Severity of Clinical Symptoms of Asthma

The severity of the severe equine asthma was assessed according to the modified Tilley et al. scale, using the scored evaluation of clinical symptoms and endoscopic examination [22]. The used scale with the scoring system is presented in Appendix A. The control group scored zero, indicating no asthma symptoms. The animals in the treated and untreated asthma groups were qualified as asthmatic patients. The results are presented in Table 1.

### 3.2. Bronchoalveolar Lavage Fluid Analysis

The cytological analysis of the bronchoalveolar lavage fluid, based on the percentage of leukocyte differential counts, showed a significantly increased number of neutrophilic cells in asthmatic horses (treated and untreated asthma groups) and a significantly higher proportion of macrophages in the control group. The results are presented in Table 2.

### 3.3. Arterial Blood Gas Analysis

The arterial blood gas analysis revealed significant differences in the partial oxygen concentration. However, the partial pressure of carbon dioxide and saturation did not differ significantly between the groups. The results are presented in Table 3.

### 3.4. Analysis of Blood Haematology

The analysis of the haematologic results from venous blood did not show statistically significant differences between the experimental groups (Appendix B, Table A2).

### 3.5. Analysis of the Venous Blood Biochemistry

The analysis of the biochemical results from venous blood did not show statistically significant differences between the experimental groups (Appendix B, Table A3).

### 3.6. Analysis of the OD280 nm Absorbance of Circulating Immune Complexes

At all the time points (T0, T1, T2, T3, T7, T14, T30), there were no significant differences between the groups. The results are presented in Table 4.

**Figure 2:**
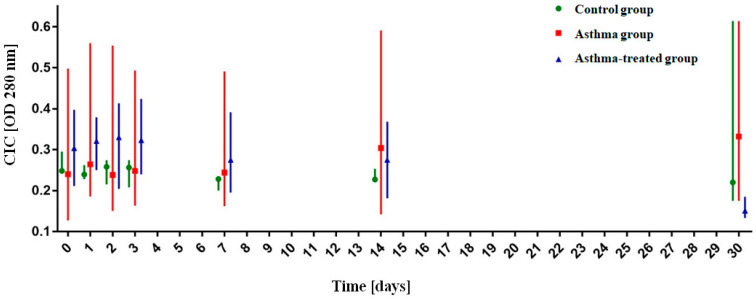
Analysis of the OD280 nm absorbance value of circulating immune complexes (CICs) over time. The graph shows the median absorbance with quartiles.

The analysis of changes over time showed that there was a significant decrease in the OD280 mm absorbance at the T30 point in the asthma-treated group from T0 median = 0.304 (q1 = 0.211, q3 = 0.397) to T30 median = 0.151 (q1 = 0.133, q3 = 0.185) (*p* < 0.001)) as well as a significant increase in the OD280 mm absorbance in T30 in the untreated group from T0 median = 0.240 (q1 = 0.127, q3 = 0.498) to T30 median = 0.332 (q1 = 0.175, q3 = 0.614) (*p* = 0.016)). In the control group, a significant decrease in the CIC was noted already at T7: from T0 median = 0.248 (q1 = 0.246, q3 = 0.295) to T7 median = 0.228 (q1 = 0.2, q3 = 0.235) with (*p* = 0.004), to T14 (*p* = 0.0302) and T30 (*p* = 0.0288). The results are presented in Figure 2.

### 3.7. The Detection of Equine IgG in Circulating Immune Complexes (CIC) Precipitated Using PEG6000

During ID and IEF analysis, the precipitating arcs of complexes formed by goat anti-equine IgG antibody and equine IgG were visible and were more distinct in samples containing elevated concentrations of CIC.

Analysis of the molecular weight of protein separated by SDS-PAGE gel electrophoresis revealed the presence of proteins whose mass corresponded to IgG antibody (MW ~164 kDa; analysis under denaturing and non-reducing condition); heavy chain of IgG antibody (MW ~49.8 kDa; analysis under denaturing and reducing condition) and light chain of IgG antibody (MW~23 kDa; analysis under denaturing and reducing condition). Immunoblotting analysis revealed the reactions with a heavy chain of IgG antibodies (data not shown).

## 4. Discussion

Research on the development and progression pathomechanisms of asthma, both in humans and in horses, is frequently conducted. This is associated not only with the economic impact of the disease on the sports and racing industry, but also with the fact that horses are a natural animal model in research on asthma in humans. The long life expectancy of these animals (30–35 years) allows for the long-term observation of sick animals, the evaluation of their inflammatory response, and the analysis of airway remodelling secondary to a naturally occurring disease, which is not seen in other animals such as mice or rats [3,25].

All the horses were divided into the experimental groups based on the results of the qualifying diagnostic procedures according to commonly used criteria. No animals with other diseases, such as orthopaedic problems, that could potentially disturb the result of the assessed parameter, were included in the study. The clinical and endoscopic scores were assessed using the modified point scale, according to Tilley et al. [22]. All the animals from the control group received a score of 0, which signified no asthma symptoms, while animals from the sick and treated group received a score of 2 or 3. All the animals underwent arterial blood gasometry to determine the partial pressure of oxygen and carbon dioxide. Significantly lower oxygen partial pressure was observed in sick animals. This observation is in accordance with reports of PaO_2_ reduction in patients with advanced asthma symptoms [1,26]. According to other authors, the partial pressure of carbon dioxide showed no significant differences between the groups [26]. Haematological and biochemical analyses of the venous blood were also performed. The results were analysed to complete data on the clinical status of the animals and to exclude coexisting diseases. There were no significant differences in the haematological and biochemical parameters of the venous blood. This is consistent with the available literature and the current inability to diagnose equine asthma using basic blood tests [2]. The animals were included in the respective groups based on the result of the cytological examination of the bronchoalveolar lavage fluid, which was based on a percentage analysis of 400 leukocytes, particularly neutrophils [21]. Due to the fact that the animals in the control group were kept in a stable system, they were considered healthy if their BALF contained up to 15% neutrophils. The maximum value obtained in that group was 13.75% [27]. The animals in the asthma group had to have a minimal neutrophil count of at least 25% of all cells. In the study, the BALF neutrophil count ranged from 39.5–87.75% of all the cells [2,27]. The animals were allocated into the treated and untreated asthma groups at random. During the diagnostic procedures we used previously described methods, which are commonly available as equine asthma diagnostic procedures [2]. We would like to perform more advanced diagnostic procedures such as those described in the literature, for example, pleural pressure measured with the oesophageal balloon catheter; however, these methods are only available in a few advanced diagnostic centres [2].

Circulating immune complexes form the basis of widespread research. Available scientific reports focus on the total number of complexes, specifically defined types of complexes such as IgE-containing complexes, as well as complex analysis after separation.

The clinical significance of the detection of circulating immune complexes has been linked to many health problems, such as allergies, autoimmune diseases, tumours, cardiac, dermatological, endocrinological, haematological nephrological, neurological, ophthalmological, parasitological, pulmonary, reproductive, transplant and infectious diseases [13].

It is believed that long-term therapy with corticosteroid drugs prolongs the circulation of immune complexes and delays their elimination in humans [28]. In the present study, no such relationship was observed in the examined horses. This may be related to the short-term treatment course with the steroid drug in the study.

In human medicine, circulating immune complexes were observed in 28% of patients with bronchial asthma and in 20% of patients with atopic asthma [28]. In other studies, the presence of complexes was reported in 39.6% of patients with bronchial asthma compared to 7.56% in the control group, with a higher frequency of detection in women than in men [29]. The mean CIC value did not differ between patients undergoing immunotherapy, untreated patients, and the control group [30]. In other studies, there were no differences in the CIC concentration in patients with bronchial asthma compared to healthy patients, although a significantly higher IgE concentration was observed in patients with asthma [31].

In children with asthma, an increase in the levels of free and bound CIC IgE with normal concentrations of IgG, IgA and IgM were observed [32]. Elevated levels of CIC were observed in 77% of patients with allergies, including 73% of non-smokers and 83% of smokers [33]. There was a higher concentration of circulating IgE-containing immune complexes in allergic patients [34].

The tests carried out on the clinically healthy horses revealed the content of CIC in the serum in the form of a mean extinction value at E450 nm. In the studied group of horses, this amounted to 0.278 (±0.096). [17]. Due to differences in methodology, the authors were not able to refer to numerical values.

In the horses with secondary arthritis, 82% had detectable CICs in serum and 77% had them in the synovial fluid. In 64% of patients, the value of CIC was elevated in both serum and synovial fluid [18]. In studies of severe equine asthma in Polish horses, a statistically higher concentration of circulating immune complexes was found in an asthma group compared to a healthy control group [20]. The lack of statistically significant differences between the groups in the present study may result from the diversity of the animals used. In the previously cited study, the animals were of one breed and kept permanently in a common environment.

In the presented study, the CIC absorbance did not differentiate patients in the control group from sick animals. It does not correlate with an earlier study performed on Polish Konik horses [20]. This may be related to the daily habitat of the animals and the difference in mean CIC levels in the particular herd from which the animals originated. In the case of the Niedźwiedź et al. study, the research groups were from one breeding stud and were kept there [20]. This would be consistent with the observation of one study, quite old, in which the mean CIC concentrations in three different studs were screened, and the results varied significantly [35]. The study on Polish Konik horses assessed the CIC levels in one time point (healthy horses vs. asthma horses during the crisis after 48 h in a dusty environment) [20]. The presented study was planned for CIC assessment over a longer period of time, and with changes of the environment as well as pharmacological treatment.

To the best of the authors’ knowledge, this is the first study to analyse the dynamics of CIC during an environmental challenge, remission, and treatment. A significant decrease in the absorbance value at the T30 time point in the treated asthma group as well as a significant increase in the absorbance value at T30 in the untreated asthma group were observed. The above results may suggest the utility of the assessment of CIC in monitoring the course of asthma. In human medicine, the usefulness of monitoring CIC levels in response to therapy in various disease entities has been noted, including Hodgkin’s lymphoma, tumours, tuberculosis and borreliosis [36,37,38]. The evaluation of this parameter requires further testing on a larger group of horses to evaluate the dynamics of CIC in response to asthma therapy. In the control group, a significant decrease in the absorbance value was noted at T7. The authors did not find any confirmation or explanation of such a situation in the literature. This may indicate the organism’s adaptability in healthy animals, but such a statement would require further research on the topic.

## 5. Conclusions

Based on the presented study, the following conclusions have been reached: the circulating immune complexes did not show straightforward utility as biomarkers in the laboratory diagnosis of equine asthma; and circulating immune complexes may become a useful parameter in monitoring the progress of asthma therapy in horses. Further research on a larger group of animals in order to determine the possibility of monitoring the progress of therapy in field practice with the help of circulating immune complexes is warranted.

## Figures and Tables

**Table 1:** The table presents the asthma severity point score of the horses. Presented are the clinical assessment—point range 0–3; airway endoscopy—point range 0–3; final score—point range 0–6; and final asthma score—point range 0–3. NS—no statistically significant differences.

	Control Group (I)			Asthma Group (II)			Asthma-Treated Group (III)			Statistical Difference (*p*)		
	q1	Median	q3	q1	Median	q3	q1	Median	q3	I and II	I and III	II and III
Clinical assessment	0.00	0.00	0.00	2.25	3.00	3.00	2.00	2.50	3.00	>0.0001	>0.0001	NS
Airway endoscopy	0.00	0.00	0.00	2.00	2.00	2.00	2.00	2.00	2.00	>0.0001	>0.0001	NS
Final score	0.00	0.00	0.00	4.25	5.00	5.00	4.00	4.50	5.00	>0.0001	>0.0001	NS
Final asthma stage	0.00	0.00	0.00	2.25	3.00	3.00	2.00	2.50	3.00	>0.0001	>0.0001	NS

**Table 2:** Percentage leukocyte differential count for bronchoalveolar lavage fluid (BALF). The median with quartiles is presented. NS—no statistically significant differences.

Cells	Control Group (I)			Asthma Group (II)			Asthma-Treated Group (III)			Statistical Difference (*p*)		
	q1	Median	q3	q1	Median	q3	q1	Median	q3	I and II	I and III	II and III
Neutrophil	5.00	7.25	12.13	55.81	78.50	86.75	49.94	63.25	72.44	0.00013	0.00105	NS
Lymphocyte	26.19	27.13	28.63	3.31	8.50	25.50	8.13	21.75	36.88	NS	NS	NS
Macrophage	60.50	62.00	62.94	8.25	11.88	16.63	10.88	13.75	17.56	0.00036	0.00081	NS
Eosinophil	0.00	0.25	0.69	0.06	0.25	3.63	0.00	0.00	0.19	NS	NS	NS
Mast cell	0.00	0.00	0.19	0.00	0.13	0.25	0.62	0.25	0.44	NS	NS	NS

**Table 3:** Median parameters of arterial blood gas analysis with quartiles. NS—no statistically significant differences.

	Control Group (I)			Asthma Group (II)			Asthma-Treated Group (III)			Statistical Difference (*p*)		
	q1	Median	q3	q1	Median	q3	q1	Median	q3	I and II	I and III	II and III
PaCO_2_	39.00	39.00	42.75	44.50	46.50	47.00	39.00	42.00	46.50	NS	NS	NS
PaO_2_	93.25	94.00	104.00	79.25	84.50	91.25	72.85	81.00	82.75	0.0019	0.0248	NS
S O2	96.25	97.00	97.00	95.25	96.50	97.00	93.35	95.00	96.00	NS	NS	NS

**Table 4:** Table showing the median OD280 nm absorbance values of circulating immune complexes with quartiles in the groups and statistical differences between groups. NS—no statistically significant differences.

Time-Point	Control Group (I)			Asthma Group (II)			Asthma-Treated Group (III)			Statistical Difference (*p*)		
	q1	Median	q3	q1	Median	q3	q1	Median	q3	I and II	I and III	II and III
T0	0.246	0.248	0.295	0.127	0.240	0.498	0.211	0.304	0.397	NS	NS	NS
T1	0.228	0.239	0.262	0.186	0.264	0.560	0.250	0.321	0.379	NS	NS	NS
T2	0.215	0.258	0.274	0.150	0.238	0.554	0.204	0.330	0.413	NS	NS	NS
T3	0.208	0.256	0.274	0.163	0.248	0.493	0.240	0.323	0.424	NS	NS	NS
T7	0.200	0.228	0.235	0.162	0.244	0.491	0.195	0.275	0.391	NS	NS	NS
T14	0.222	0.227	0.253	0.142	0.304	0.591	0.181	0.275	0.368	NS	NS	NS
T30	0.175	0.220	0.614	0.175	0.332	0.614	0.133	0.151	0.185	NS	NS	NS

## Appendix A. The Modified Tilley Scores

**Table A1:** The modified Tilley scores [22].

Parameter	0	1	2	3	4	5
Clinical Assessment ^a^						
*Cough score*	None	Coughs at specific times of day (feeding/exercising/making beds)	Frequent cough with periods of no coughing	Very frequent cough		
*Nostril flare*	None	Flares during inspiration (returns to normal at end inspiration)	Flares on inspiration and exhalation (slight movement can still be seen)	Flares on inspiration and expiration (no movement can be seen)		
*Abdominal lift*	None	Slight flattening of ventral flank	Obvious abdominal flattening and “heave line” extending no more than halfway between cubital joint and *tuber coxae*	Obvious abdominal lift and “heave line” extending beyond halfway between cubital joint and *tuber coxae*		
Airway endoscopy ^b^						
*Mucus accumulation*	None, clean	Few, multiple small blobs	Moderate, larger blobs	Marked, confluent or stream-forming	Large, pool- forming	Extreme, profuse amounts
*Mucus color*	None, clean	Colorless	White	Thick White	Yellow	Thick Yellow
*Mucus localisation and stickiness*	None, clean	1/2 Ventral	2/3 Lateral	3/4 Dorsal	Threading	Threading
*Mucus apparent viscosity*	None, clean	Very fluid	Fluid	Intermediate	Viscous	Very viscous

^a^ Final Clinical Score (CS): 0 (CS final score < 2); 1 (2 ≤ CS final score ≤ 4); 2 (5 ≤ CS final score ≤ 6); 3 (7 ≤ CS final score ≤ 9); ^b^ Final airway Endoscopy Score: 0 (ES final score < 8.5); 1 (8.5 ≤ ES final score ≤ 12); 2 (12 < ES final score ≤ 16); 3 (ES final score > 16).

Final Severe Asthma Stage:

Stage 0—No Asthma (Total Score = 0);

Stage 1—Severe Asthma (1 ≤ Total Score < 2);

Stage 2—Severe Asthma (3 ≤ Total Score ≤ 4);

Stage 3—Severe Asthma (5 ≤ Total Score = 6)

## Appendix B. The Analysis of the Haematologic and Biochemical Results from Venous Blood

**Table A2:** Median parameters of haematologic blood analysis with quartiles. NS—no statistically significant differences.

Parameter	Control Group (I)			Asthma Group (II)			Asthma-Treated Group (III)			Statistical Difference (*p*)		
	q1	Median	q3	q1	Median	q3	q1	Median	q3	I and II	I and III	II and III
White Blood Cells (10^9^/L)	6.00	7.05	8.33	7.35	7.65	7.95	6.60	6.85	7.78	NS	NS	NS
Red Blood Cells (10^12^/L)	8.14	8.95	9.76	6.50	7.50	8.55	6.85	7.69	8.21	NS	NS	NS
Heoglobin (mmol/L)	7.43	7.90	8.53	7.00	7.65	8.45	7.08	7.35	7.93	NS	NS	NS
Hematocrit (l/L)	0.36	0.38	0.42	0.33	0.37	0.42	0.32	0.34	0.35	NS	NS	NS
Platelet count (10^9/L)	143.00	186.50	230.00	159.50	183.50	208.25	115.25	138.00	160.00	NS	NS	NS
Mean Corpuscular Hemoglobin (fmol/L)	46.00	47.00	48.75	49.00	50.00	51.00	43.50	45.00	4500	NS	NS	NS
Mean Corpuscular Hemoglobin Concentration (mmol/L)	20.23	20.50	20.70	20.33	20.60	20.80	20.75	20.90	20.98	NS	NS	NS
Red cell Distribution Width (%)	16.55	16.90	17.48	16.95	17.10	17.10	16.55	16.90	17.48	NS	NS	NS
Mean Platelet Volume (fL)	5.63	5.75	5.88	5.93	6.00	6.30	5.58	6.05	6.60	NS	NS	NS
Lymphocytes (10^9^/L)	1.68	2.20	2.73	1.15	1.60	2.13	1.43	1.55	2.13	NS	NS	NS
Monocytes (10^9^/L)	0.33	0.40	0.48	0.33	0.40	0.55	0.30	0.30	0.30	NS	NS	NS
Granulocytes (10^9^/L)	3.73	4.25	5.00	4.93	5.55	6.48	4.73	5.05	5.38	NS	NS	NS

**Table A3:** Median parameters of biochemical blood analysis with quartiles. NS—no statistically significant differences.

Parameter	Ref. Value	Control Group (I)			Asthma Group (II)			Asthma-Treated Group (III)			Statistical Difference (*p*)		
		q1	Median	q3	q1	Median	q3	q1	Median	q3	I and II	I and III	II and III
Aspartate aminotransferase (ASPAT)	205–555 U/L	278.00	292.50	310.75	247.25	278.00	290.00	240.50	286.50	369.25	NS	NS	NS
Alanine aminotransferase (ALAT)	3–25 U/L	5.25	6.50	7.75	4.00	4.00	4.75	5.00	5.50	9.00	NS	NS	NS
Gamma-Glutamyl Transferase (GGT)	12–45 U/L	9.25	10.50	11.00	12.00	12.50	14.50	9.50	11.50	14.25	NS	NS	NS
Creatine kinase (CK)	90–565 U/L	204.75	249.00	299.25	214.50	264.50	278.50	136.00	185.50	259.75	NS	NS	NS
Total Bilirubin (BILI-T)	13.7–25.6 µmol/L	18.23	21.40	25.55	19.70	22.00	24.98	17.30	25.00	35.33	NS	NS	NS
Urea	4.2–7.5 mmol/L	4.90	5.80	6.10	4.23	4.70	5.25	4.43	4.75	5.08	NS	NS	NS
Creatinine	106–168 µmol/L	128.00	133.00	139.50	108.50	113.50	117.00	127.00	127.00	133.75	NS	NS	NS
Albumin	25–59 g/L	33.00	33.00	33.75	31.75	34.00	34.00	31.25	32.00	32.00	NS	NS	NS
Tolal Protein	60–78 g/L	59.00	59.50	66.00	59.00	65.00	66.50	65.50	68.50	70.00	NS	NS	NS
Glucose	3.1–6.2 mmol/L	3.55	4.10	5.18	4.73	6.05	6.18	4.58	5.10	5.55	NS	NS	NS
Calcium	2.25–3.12 mmol/L	3.03	3.13	3.15	2.95	3.04	3.16	3.21	3.22	3.40	NS	NS	NS
Iron	18–35 µmol/L	20.50	21.30	26.83	20.70	23.90	25.60	21.30	22.45	23.38	NS	NS	NS
Sodium	139.1–156.5 mmol/L	131.48	132.75	135.15	133.33	134.80	136.65	136.68	137.10	137.53	NS	NS	NS
Potassium	3.5–4.7 mmol/L	4.31	4.73	4.79	3.87	4.03	4.15	3.06	3.39	3.50	NS	NS	NS
Chlorides	90.2–107.2 mmol/L	99.48	100.90	101.73	95.98	99.40	100.88	99.35	99.55	100.80	NS	NS	NS
Magnesium	0.7–1.15 mmol/L	0.86	0.89	0.92	0.68	0.71	0.78	0.85	0.88	0.90	NS	NS	NS
Bile Acids	µmol/L	1.63	1.80	2.10	3.55	4.45	5.75	4.45	5.80	7.53	NS	NS	NS
